# Pharmacoepigenomics in Personalized Medicine: A Hypothesis-Generating Approach to Introduce CpG-PGx SNPs as New Candidates for a Systematic Insight into Genomic-Epigenomic-Phenomic-Pharmacogenomics (G-E-Ph-PGx) Axis

**DOI:** 10.3390/jpm15120579

**Published:** 2025-11-29

**Authors:** Alireza Sharafshah, Kenneth Blum, Kai-Uwe Lewandrowski, Igor Elman, Brian S. Fuehrlein, David Baron, Albert Pinhasov, Panayotis K. Thanos, Rossano Kepler Alvim Fiorelli, Sergio L. Schmidt, Eliot L. Gardner, Morgan P. Lorio, Alexander P. L. Lewandrowski, Mark S. Gold

**Affiliations:** 1 Cellular and Molecular Research Center, School of Medicine, Guilan University of Medical Sciences, Rasht 4144666949, Iran; 2 The Kenneth Blum Behavioral & Neurogenetic Institute, Austin, TX 78701, USA; business@tucsonspine.com (K.-U.L.); dbaron@westernu.edu (D.B.); 3 Institute of Psychology, ELTE Eötvös Loránd University, H-1117 Budapest, Hungary; 4 Department of Molecular Biology, Adelson School of Medicine, Ariel University, Ariel 40700, Israel; dr.igorelman@gmail.com (I.E.); albertpi@ariel.ac (A.P.); thanos@buffalo.edu (P.K.T.); 5 Center for Psychiatry and Sport, Western University Health Sciences, Pomona, CA 91766, USA; 6 Division of Personalized Medicine, Center for Advanced Spine Care of Southern Arizona, Tucson, AZ 85712, USA; 7 Programa de Pós-Graduação em Neurologia, Universidade Federal do Estado do Rio de Janeiro, Rio de Janeiro 20270-004, Brazil; fiorellirossano@hotmail.com (R.K.A.F.); slschmidt@terra.com.br (S.L.S.); 8 Department of Spine Surgery, School of Medicine, University of Arizonia, Tucson, AZ 85724, USA; 9 Department of Psychiatry, Cambridge Alliance, Harvard University School of Medicine, Cambridge, MA 02215, USA; 10 Department of Psychiatry, Yale University School of Medicine, New Haven, CT 06511, USA; brian.fuehrlein@va.gov; 11 Department of Psychiatry, Stanford University School of Medicine, Palo Alto, CA 94305, USA; 12 Behavioral Neuropharmacology and Neuroimaging Laboratory on Addictions (BNNLA), Clinical Research Institute on Addictions, Department of Pharmacology and Toxicology, Jacobs School of Medicine and Biomedical Sciences, University at Buffalo, Buffalo, NY 14260, USA; 13 Neuropsychopharmacology Section, Intramural Research Program, National Institute on Drug Abuse, National Institutes of Health, Baltimore, MD 20892, USA; egardner@intra.nida.nih.gov; 14 Advanced Orthopedics, 499 E. Central Pkwy, Ste. 130, Altamonte Springs, FL 32701, USA; mloriomd@gmail.com; 15 Department of Biological Sciences, Dornsife College of Letters, Arts and Sciences, 3616 Trousdale Pkwy, Los Angeles, CA 90089, USA; lewandro@usc.edu; 16 Department of Psychiatry, Washington University School of Medicine, St. Louis, MO 63110, USA; mgold9876@wustl.edu

**Keywords:** Pharmacoepigenomics, CpG-PGx SNP, CpG site, CYP2D6, TET2

## Abstract

**Background**: There are important gaps in describing the associations between variants found by GWAS and various phenotypes. Prior reports suggest that SNPs in regulatory regions should be further investigated to uncover these associations. Thus, this study involved a novel approach, along with Pharmacoepigenomics, prompting a new coined term “CpG-PGx SNP”. **Methods**: The rationale behind our analysis strategy was based on the impact of SNPs playing dual roles both in the CpG site disruption/formation and having PGx associations. Thus, we employed GeneCards (relevance score), PharmGKB (significant *p*-value), and GWAS catalog data for each gene (*p* < 5 × 10^−8^). Following the obtainment of the 25 best-scored genes of four major epigenetic processes (methylation, demethylation, acetylation, and deacetylation), we generated two lists of candidate genes, including potential CpG-PGx SNPs and possible CpG-PGx SNPs. Results: Among 2900 significant PGx annotations, we found 99 potential CpG-PGx SNPs related to 16 genes. *CYP2B6*, *CYP2C19*, *CYP2D6*, and *COMT* genes were the top genes. Additionally, we found 1230 significant GWAS-based SNPs, among them 329 CpG-SNPs related to 48 genes with at least one CpG site disruption/formation. The top gene with the highest CpG-SNPs was *TET2*, followed by *JMJD1C* and *HDAC9*. Importantly, we detected some synonymous variants in the Epigenetically Modifiable Accessible Region (EMAR), which can provide insights into undiscovered roles of these SNPs. We identified 173 CpG-Disruptive SNPs, 155 CpG-Forming SNPs, and just 1 CpG SNP with both impacts. **Conclusions**: In conclusion, here we introduce CpG-PGx SNP for the first time and suggest three major genes playing crucial roles in Pharmacoepigenomics (PEpGx), *CYP2D6* as the heart of PEpGx, and *TET2* with the highest possibility of having CPG-PGx SNPs. We believe that this approach will help the scientific community to utilize “CpG-PGx SNP” to unravel complex disease-driven genetic and epigenetic interactions, yielding therapeutic opportunities.

## 1. Introduction

Numerous traits have been effectively linked to specific areas of the genome by genome-wide association studies (GWAS) [1]. The process by which variations impact the phenotype that they are linked to is still unclear for many of these observations [2]. The majority of trait-associated variations found by GWAS are thought to function by changing gene expression rather than changing the protein coding, and are found in regulatory areas of the genome [3]. This hypothesis is supported by the discovery of overlaps between GWAS risk variants and genomic loci influencing markers of genome regulation (like histone modifications) and enrichment of expression quantitative trait loci (eQTLs) at identified GWAS risk loci [4,5,6,7]. Therefore, combining GWAS with gene expression data is one plausible way to improve knowledge of the processes related to GWAS findings.

DNA methylation is a key process in gene regulation. As such, it is an essential intermediary molecular trait that connects genes to other macro-level phenotypes and may contribute to missing heritability [8]. Despite their physiological importance, the genetic drivers of DNA methylation patterns remain poorly understood. There is evidence that genetic variation at certain loci correlates with the quantitative characteristic of DNA methylation [9,10,11,12]. Additionally, previous studies discovered that genetic variants at CpG sites (meSNPs) can possibly disrupt the substrate of methylation reactions and thus, severely alter the methylation status at a single CpG site [13,14].

While methylation-associated single-nucleotide polymorphisms (meSNPs) have been identified in various studies, it remains unclear if meSNPs constitute a major class of methylation quantitative trait loci (meQTLs), or if they significantly influence the methylation status of nearby CpG sites [15,16,17,18]. Most meQTL studies to date have been limited by relatively small sample sizes and the use of low-resolution methylation microarrays, in which meSNPs are sparsely represented. Furthermore, many current meQTL analyses deliberately exclude probes overlapping with sequence variants to avoid confounding due to disrupted probe hybridization [15,16].

Pharmacoepigenetics explores the complex relationship between epigenetic modifications and pharmacological responses, emphasizing how drugs can both alter and be affected by epigenetic mechanisms [19,20]. Gaining insight into these epigenetic changes is essential in pharmacology, as it could help optimize drug efficacy, reduce adverse reactions, and drive forward the progress of personalized medicine. As a multidisciplinary and continuously evolving field, pharmacoepigenetics merges pharmacology, epigenetics, and other life sciences to shape innovative therapeutic strategies and uncover new drug targets [21]. Alongside pharmacogenomics—a pharmacological sub-discipline focused on genetic variability in drug response—Pharmacoepigenomics has emerged as a key area of interest. It concentrates on epigenetic therapy, the impact of epigenetic regulation on pharmacokinetics, and its implications in adverse drug reactions [22].

Finally, both pharmacoepigenetics and pharmacogenomics play a vital role in advancing personalized medicine by shedding light on the intricate relationships between genes, epigenetic mechanisms, and drug responses [22]. Here, we introduce an innovative idea to test the Genomic-Epigenomic-Phenomic-Pharmacogenomics (G-E-Ph-PGx) axis by employing CpG-PGx SNPs (whose PGx roles are known) and possible CpG-PGx SNPs.

## 2. Materials and Methods

### 2.1. General Design

**Figure 1:**
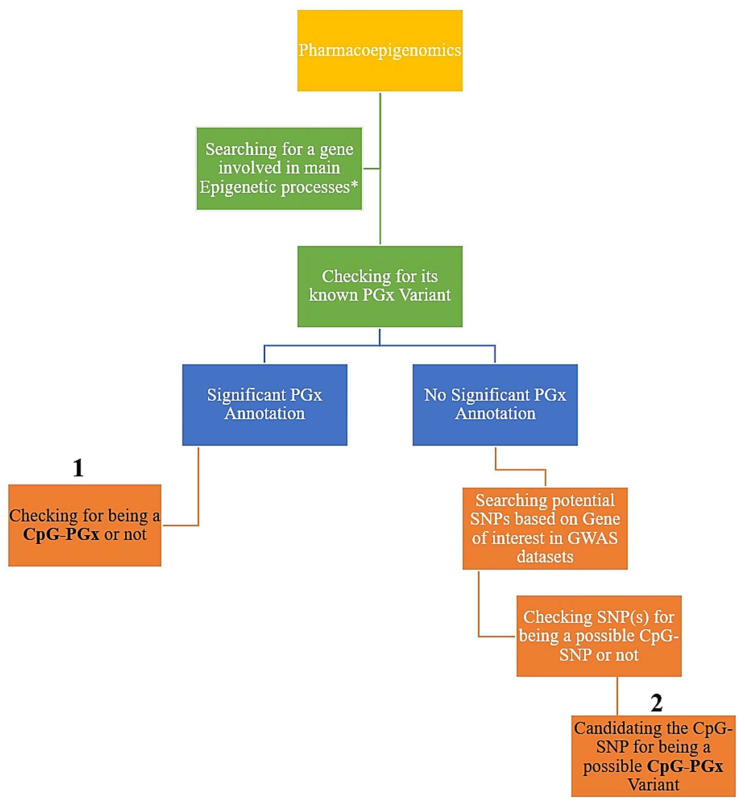
Pharmacoepigenomics is summarized in a hierarchal flow. Main epigenetic processes (*) depicted indicate methylation, demethylation, acetylation, and deacetylation. Numbers 1 and 2 refer to the two plausible ways to delineate a CpG-PGx SNP, whereby number 1 indicates an easier way than number 2. However, number 2 could also introduce new CpG-PGx SNP(s) compared with number 1. It is noteworthy that GeneCards, PharmGKB, and the GWAS catalog are employed in this design.

Firstly, we analyzed the four major processes in epigenetics, including methylation, demethylation, acetylation, and deacetylation. Then, we searched for the best-scored genes in each epigenetic process according to the relevance score of GeneCards (access date: 1 July 2025, https://www.genecards.org/) [23]. Accordingly, we calculated the 1st–25th best-scored genes for each of the 4 epigenetic processes. Secondly, we checked every 100 top genes in PharmGKB (access date: 1 July 2025, now ClinPGx available at: https://www.clinpgx.org/) [24] to see if they had at least one significant PGx annotation. Thirdly, each PGx variant was subsequently checked for possible categorization as a CpG-PGx SNP. To accomplish this, we employed the Ensembl genome browser with reference genome GRCh38; more specifically, we manually searched the rsID for each SNP in Ensembl (Ensembl 114 corresponding release of Ensembl Genomes 61). We also checked the major allele, a nucleotide pre-and post to determine its location relevant to a CpG dinucleotide. Following this event, we checked the minor allele accordingly. If the major allele was in a CpG dinucleotide, then it would be a CpG-SNP, which could be disrupted by a minor allele, and if the major allele shaped a new CpG-SNP, then it could be considered as a forming CpG site. Ensembl was also utilized to determine SNP functions, including exonic (missense or synonymous), intronic (regulatory), upstream (5′UTR), and downstream (3′UTR). It is indeed noteworthy that the classification of regions linking SNPs was directly obtained from Ensembl (access date: 1 July 2025, https://www.ensembl.org/index.html), such as Epigenetically Modifiable Accessible Region (EMAR), Promoter, Enhancers, and CTCF Binding Sites (CBS). Thus, these regions were automatically displayed by zooming the sequential viewer. To determine any newly found CpG-PGx SNP based on our hypothesis-generating approach, we investigated genes that had no significant PGx annotation in the GWAS catalog (access date: 1 July 2025, https://www.ebi.ac.uk/gwas/home) [25]. Finally, we checked the potential SNPs (based on the best *p*-values) for possible categorization as a CpG SNP. These CpG SNPs were designated in the Section 3 as new CpG-PGx SNPs. The whole process adhered to a hierarchal flow which provided any potential role SNPS of PGx annotations in both epigenetic processes or as a pharmacoepigenetic factor (Figure 1).

Applying GeneCards, we included 100 best-scored genes based on 4 epigenetic processes, including methylation, demethylation, acetylation, and deacetylation (25 top genes of each one). It is noteworthy that all of the included genes were protein-coding due to the major interactions of protein–drugs in real-world findings. As such, this provided high confidence in our introduction of new CpG-PGx SNP(s) for future confirmations. As a well-known dataset, we established our strategy utilizing PharmGKB information regarding its basis, consisting of CPIC and DPWG as its main pillars. Finally, to further confirm the new CpG-PGx SNPs, we utilized the GWAS catalog for a gene of interest and refined the potential SNPs based on its classified data.

### 2.2. Statistical Analysis

According to our analysis strategy, we prioritized and filtered genes, PGx annotations, and CpG-SNPs related to various statistical scores. Firstly, we utilized GeneCards data for determining the top genes in 4 epigenetic processes (methylation, demethylation, acetylation, and deacetylation) based on Elasticsearch 7.11, including the Relevance score. The theory behind Relevance Scoring is that Lucene (and thus Elasticsearch) utilizes the Boolean model to find matching documents, and a formula termed “the practical scoring function” to compute relevance. This formula, itself, borrows concepts from the term frequency/inverse document frequency and the vector space model; however, it adds more-modern characteristics such as a field length normalization, coordination factor, and term/query clause boosting. Importantly, supplementary boosting is provided for the annotations, including the Symbol, Aliases, and Descriptions, Accessions for the major bioinformatics databases (NCBI, Ensembl, SwissProt), Molecular function(s), Gene Summaries, Variants with Clinical Significance, and Elite disorders. Additionally, we also employed PharmGKB, which is a pharmacogenomics resource that incorporates clinical data, including clinical guidelines and medication labels, associations of potentially clinically actionable gene-drug, and genotype-phenotype linkages. To note, PharmGKB collects, curates, and publicizes knowledge regarding the effect of human genetic variation on drug responses. This is accomplished via several activities such as annotating the genetic variants and gene–drug–disease relationships via literature review: summarizing the vital pharmacogenomic genes, associations between genetic variants and drugs, and drug pathways, and curating FDA drug labels covering pharmacogenomic data. The main filtering step in PharmGKB actually considered a significant *p*-value of lower than 0.05 for all obtained PGx annotations. Finally, we mined a number of genes generating the primary list (remained/extracted from step 1) in the GWAS catalog, and also adjusted the False Discovery Rate (FDR). Moreover, we considered the critical threshold of *p*-value < 5 × 10^−8^. Thus, we included the most significant GWAS-based SNPs in the current study to increase the validation of our predictions and narrow the possibilities to be close to future real-world findings.

## 3. Results

As we described earlier in the Method Section, the aim of this study is to provide new prospects for personalized medicine treatment by advancing PGx approaches. We believe that SNPs, as the smallest genetic building blocks, can have major impact by playing multiple roles and have the potential to induce important changes by additive functions (SNP-SNP interactions) [26]. Pharmacoepigenomics can simultaneously be elucidated by CpG-SNPs having PG roles, and as such, we divided the data into augmented detail of the primary genes (100 genes having 4 major epigenetic impacts).

Initially, we obtained only the best-scored protein-coding genes from GeneCards for each of the 4 epigenetic processes. This was accomplished following a precise search in PharmGKB. Thus, we separated primary genes with at least 1 significant PGx annotation from genes with no PGx annotation archived in PharmGKB. This separation aligned with the two possible ways of finding CpG-PGx SNPs represented in Figure 1. It should be clearly noted that a unique SNP may have one or more than one PGx annotations. A PGx annotation refers to a Variant-Drug-Association.

### 3.1. Potential CpG-PGx SNPs

Table 1 summarizes the primary genes with epigenetic impact that have at least one significant PGx annotation based on PharmGKB. Accordingly, 22 unique genes out of 100 primary genes represented significant PGx annotation(s); notably, *TP53*, *HDAC1*, *KAT2B*, and *SIRT1* were duplicated. *TP53* is involved in methylation, acetylation, and deacetylation; *HDAC1*, *KAT2B*, and *SIRT1* are involved in the acetylation and deacetylation process. The top Pharmacogene based on Table 1 was *CYP2C19* with 949 significant PGx annotations; also, *CYP2D6* and *CYP2B6* were the second and third best-scored Pharmacogenes with 733 and 383 significant annotations, respectively. Interestingly, *COMT* was the seventh best-scored Pharmacogene with 121 PGx annotations. Secondly, we searched each annotation for a potential CpG-PGx SNP. Table 1 has a separate column showing this potential. Accordingly, the top gene based on the number of CpG-PGx SNPs was *CYP2B6* with 23 CpG-PGx SNPs, followed by *CYP2C19* with 21 CpG-PGx SNPs and *CYP2D6* with 18 CpG-PGx SNPs. Remarkably, all of these genes are involved in the demethylation process, and *COMT* (11 CpG-PGx SNPs) showed the top-scored gene amongst those that are involved in the methylation process. Finally, 16 genes out of 22 genes were revealed to have potential CpG-PGx SNPs (Table 1).

Thirdly, we focused on each CpG-PGx SNP to check its function (Missense, Synonymous, Intronic, Spicing, 3′UTR, 5′UTR, or being in the regulatory region, e.g., Enhancer). To do this, we checked each CpG-PGx SNP in the Genome Browser via Ensembl for its major and minor alleles. This was accomplished for finding the CpG site formation or disruption by allelic change. It is noteworthy that this is a vital check to introduce a CpG-PGx SNP for further investigations. Specifically, CpG site formation is basically hidden in the Genome Browser. However, a minor allele is denoted as a C in a dinucleotide of XpG (where X can be A, T, or G allele) or a G in a dinucleotide of CpY (where Y can be A, C, or T allele). On the other hand, CpG site disruption is derived from a SNP in either C or G of a CpG dinucleotide site (actually, there might be ApG, TpG, GpG, CpA, CpT, or CpC dinucleotides). Table 2 verifies each CpG-PGx SNP (based on known rsIDs) and its related gene, function, and CpG site situation. Generally, we found 99 CpG-PGx among them, 61 variants were missense variants, 25 variants were Intronic, 4 variants were 3′UTR, 4 variants were in a regulatory feature, 3 variants were Synonymous, 1 variant was a Spicing, and 1 variant was a Frameshift (Table 2). *CYP2D6* indicated a range for having various types of variants, including missense, intronic, splicing, frameshift, and missense/inframeshift variants.

### 3.2. The Heart of Pharmacoepigenomics?

Based on the well-known data in the PGx literature, *CYP2D6* is involved in the metabolism of almost 25% of commonly used drugs [27] and here, following intense investigation, we suggest it as the heart of pharmacogenetics, but this should be further investigated. However, our findings suggested *CYP2B6* as the top gene based on the number of CpG-PGx SNPs. To reach a more precise comparison among the three best-scored genes, including *CYP2B6*, *CYP2C19*, and *CYP2D6*, we considered more factors, including relevance score (obtained from GeneCards), number of significant PGx annotations (Obtained from PharmGKB), CpG-PGx SNPs (presented in Table 1), type of variants (based on the related SNP functions in Table 2), Number of CpG site formations, Number of CpG site disruptions, and including the title of papers indexed in PubMed (Table 3). *CYP2D6* showed the best factors (4 out of 7), including the best relevance score (10.74248), having the most types of variants (5), the highest number of CpG site formation (10), and highly impactful indexing (2658 papers in their titles). Thus, we strongly suggest that the other two genes (*CYP2C19* and *CYP2B6*) represent potential candidates for being the hub genes of Pharmacoepigenomics.

### 3.3. Putative CpG-PGx SNPs

**Figure 2:**
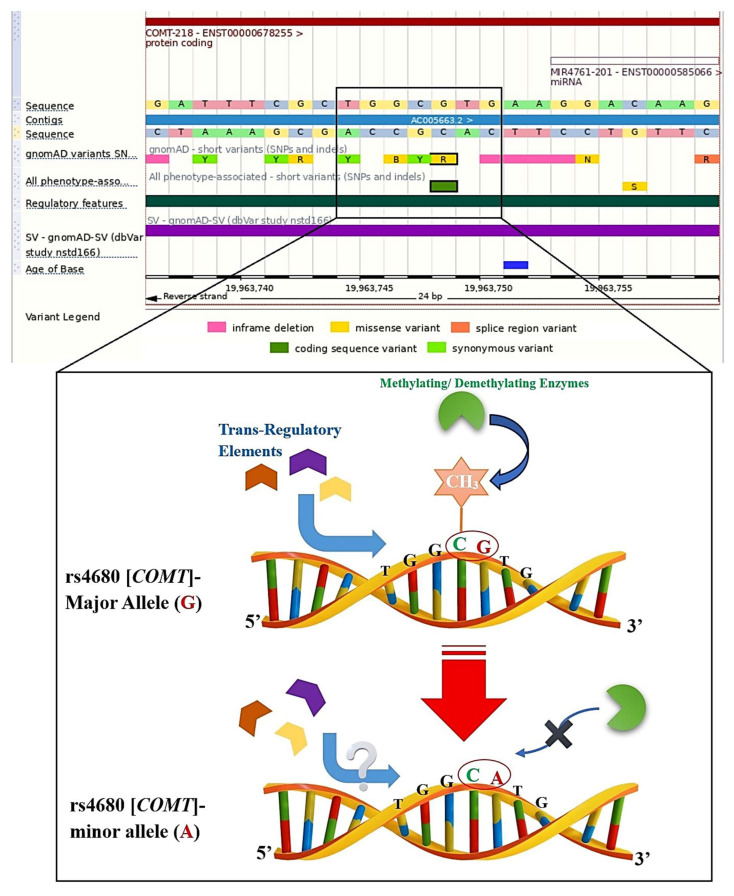
Schematic overview of our basic methodology in finding the CpG-SNPs and then checking them for their type concerning CpG forming or CpG disrupting. In this illustration, we selected rs4680, which is the well-known SNP of the COMT gene, to show the possibilities. As it is clear, in the first step, we searched each SNP of interest (here rs4680) in the Ensembl genome browser and then checked its sequence for being in a CpG site or not; moreover, if a SNP was not in a CpG site, we further checked the minor allele for finding whether it might be a forming CpG site or not. Notably, in this figure, we indicate that methylation-associated enzymes are involved with the methyl group of CpG site (CH3), and this process may have an influence on the binding affinities of trans-regulatory elements, which, in turn, these whole biological processes can potentially be interrupted in the CpG disruption by changing an allele (here, major to minor).

Delving into deeper layers of PEpGx, we extracted the remaining genes with no significant PGx annotation and thereafter. By mining the related data of these genes in the GWAS catalog, we refined the significant SNPs with a *p*-value lower than 5 × 10^−8^ and Minor Allele Frequency (MAF) of higher than 0.05. Finally, we checked the resultant SNPS in the Genomic Region browser (access date: 1 July 2025, https://www.ensembl.org/index.html?redirect=no) to determine (1) if they can form a new CpG site (CpG forming) or (2) disrupt a present CpG site (CpG Disruptive) (Figure 2).

The final putative CpG-SNPs were checked for their PGx associations to confirm whether each of the CpG-SNPs would constitute its categorization of being a CpG-PGx SNP. To note, this is indeed the second pathway described in Figure 1. According to the results indicated in Table 4, for all of the remaining 69 genes (some genes were involved in more than one epigenetic process), we mined 1,230 significant GWAS associations (or SNPs), which revealed 329 CpG-SNPs related to 48 genes with at least one CpG site formation or disruption. The top gene with the highest CpG-SNPs was *TET2* (42 CpG-SNPs), followed by *JMJD1C* (35 CpG-SNPs) and *HDAC9* (26 CpG-SNPs) in the second and third places, respectively. Interestingly, the demethylation process was not only the most important process, but also demethylation was present in the second, third, fourth, and fifth places. The other most important process was methylation by *GRIN2A* (13 CpG-SNPs).

Moreover, in the next step, we separated the CpG-Disruptive SNPs from CpG-Forming SNPs. In total, we found 173 CpG-Disruptive SNPs, 155 CpG-Forming SNPs, and just 1 CpG SNP with both disruptive and forming impact (it can be between 2 CpG sites and disrupts one and forms the second one as a new CpG site). One example we found was the intronic SNP (rs34770920) of the *ACAA2* gene. Furthermore, we found an interesting epigenetic impact in synonymous SNPs, which agrees with our previous result in the Section 3.1. More specifically, we found some CpG-SNPs in the EMAR, such as rs10849885 (*KDM2B*; synonymous; MAF: 0.5; CpG-Disruptive SNP), rs1667619 (*TET3*; synonymous; MAF: 0.47; CpG-Disruptive SNP), rs601999 (*NAGLU*; synonymous; *MAF*: 0.29; CpG-Forming SNP), and rs591939 (*NAGLU*; synonymous; MAF: 0.18; CpG-Forming SNP). We hereby propose that these synonymous CpG-SNPs cannot change the amino acid sequence as well as the protein structure (having no visible impact on the “Human Genome”), but they still represent a CpG-Forming SNP and are involved in the regulatory mechanisms.

Finally, in the last step, we attempted to classify both CpG-Disruptive SNPs and CpG-Forming SNPs based on their MAFs. As such, it is noteworthy to point out the undeniable rule of statistical and epidemiological genetics, which defines the possibilities of carrying SNPs by either an individual or various general populations. In Table 5 and Table 6, all CpG-SNPs are presented from the most to the least common, along with their functions. In this regard, there were nine CpG-Disruptive SNPs, including one with the MAF of 0.5 (the highest prevalence) and four CpG-Disruptive SNPs with the MAF of 0.05 (the smallest prevalence).

The most prevalent CpG-Disruptive SNPs were rs2984348 (*HDAC8*; Enhancer), rs13245206 (*HDAC9*; Intronic); rs10237149 (*HDAC9*; Intronic), rs6951745 (*HDAC9*; Intronic), rs10849885 (*KDM2B*; Synonymous/EMAR), rs12001316 (*KDM4C*, Intronic), rs3814177 (*TET1*; 3′UTR), rs9884984 (*TET2*; Intronic); and rs6533183 (*TET2*; Intronic) (Table 5 and Supplementary Table S1).

Accordingly, in the group of CpG-Forming SNPs, we found 13 CpG-SNPs with the highest prevalence (MAF = 0.5) and 3 CpG-SNPs with the lowest prevalence (MAF = 0.05). The most prevalent CpG-Forming SNPs included rs1931537 (*AR*; 3′UTR), rs2116942 (*DNMT1*; Missense), rs1935 (*JMJD1C*; Missense), rs7962128 (*KDM2B*; 3′UTR), rs6489811 (*KDM2B*; Intronic), rs2613766 (*KDM4B*; Intronic), rs7042372 (*KDM4C*; Intronic/EMAR/Enhancer), rs960658 (*KDM4C*; Intronic), rs7037266 (*KDM4C*, Intronic), rs5969750 (*RBBP7*; 3′UTR), rs7670522 (*TET2*; 3′UTR), rs9884296 (*TET2*; Intronic), and rs5952279 (*KDM6A*; Intronic) (Table 6 and Supplementary Table S2).

## 4. Discussion

To the best of our knowledge, this is the first hypothesis-generating approach introducing CpG-PGx SNP as a multi-dimensional candidate in a Genomic-Epigenomic-Phenomic-Pharmacogenomics (GEPh-PGx) axis. GEPh-PGx suggests a complicated network of regulatory-functional interactions initiated from the smallest genetic block (SNP) to the broader cellular and molecular interplay leading to known and unknown phenotypes, which, in turn, are linked to pharmacological interactions and treatments. Briefly, GEPh-PGx represents a new aspect of personalized medicine based on the disruption or formation of a CpG site by allele changes in an SNP. This phenomenon clearly helps explain the trans-regulation processes in which these CpG sites can present or remove the possible epigenetic tags for Methylation/Demethylation reactions.

We designed a logical and comprehensive strategy of analysis based on the well-known and documented list of various genes in all four classical epigenetic processes, including methylation, demethylation, acetylation, and deacetylation. In the current investigation, we mined the CpG sites for all these genes involved in methylation/demethylation and also included genes for acetylation and deacetylation processes. Therefore, we selected 100 genes and, following the removal of the duplications (some genes were present in more than one epigenetic process, like *TP53*), 91 unique genes remained. We followed two pathways, including searching for and introducing potential CpG-PGx SNPs and possible CpG-SNPs to be newly confirmed CpG-PGx SNPs. We found 3 major genes for having the highest number of potential CpG-PGx SNPs, including *CYP2B6*, *CYP2C19*, and *CYP2D6*. Among them, *CYP2D6* was found to be the heart of Pharmacoepigenomics. Finally, after a deep search based on GWAS data, we found *TET2* as the top-scored candidate for future PGx confirmations according to its number of possible CpG-SNPs.

There are some studies concerning CpG-SNP(s) directly in their titles (26 papers in PubMed) and also in their abstracts (20 papers in PubMed). All the PubMed-indexed papers for CpG-SNPs in their titles can be divided into three main categories, including Neuropsychological disorders, such as suicidal behavior in subjects with schizophrenia [28], psychosis [29] and major depressive disorder [30], metabolic disorders, including type 2 diabetes [31,32] and obesity [33,34], and cancer biology [35].

Pharmacoepigenetics and Pharmacoepigenomics revealed a better resulting outcome compared with CpG-SNPs in the literature. We found 24 papers in PubMed with the pharmacoepigenetics or Pharmacoepigenomics linked in their titles. Interestingly, similar to the aforementioned 3 major categories, these papers focused on the same categories. Montagna was one of the first scientists who discussed the epigenetic and pharmacoepigenetic processes in primary headaches and pain [36]. Leach et al. reviewed pharmacoepigenetics in heart failure and cardiovascular disease (CVD) and concluded that, because epigenetics has a vital role in shaping phenotypic variation in health and disease, understanding and manipulating the epigenome has a massive capacity for the treatment and prevention of common human diseases [37]. In the context of cancer, Candelaria et al., with an emphasis on gemcitabine, reviewed an update of genetic and epigenetic bases that might account for inter-individual variations in therapeutic results [38]. Accordingly, Nasr et al. studied pharmacoepigenetics in breast cancer [39], Fornaro et al. reviewed pharmacoepigenetics in gastrointestinal cancer [40], and Gutierrez-Camino et al. reported on pharmacoepigenetics in childhood acute lymphoblastic leukemia [41]. In a meta-analysis, Chu and Yang systematically studied the population diversity impact of DNA methylation on the treatment response and drug ADME in various tissues and cancer types. They concluded that ethnicity should be cautiously considered for future pharmacoepigenetics explorations [42]. Notably, Nuotio et al. performed a genome-wide methylation analysis of responsiveness to four classes of antihypertensive drugs in the pharmacoepigenetics of hypertension [43].

The last and most important topic in pharmacoepigenetics is psychological and behavioral phenotypes, such as generalized anxiety disorder [44], Alzheimer’s disease [45], and depression [46], and opioid addiction [47].

Epigenetic variants have been found near genes and gene regulators, which control the metabolism of drugs, suggesting a role for epigenetic mechanisms in modulating pharmacokinetics and pharmacodynamics [48,49,50]. Pharmacoepigenetics is a field that studies how epigenetic variability impacts variability in drug response [20]. Of note, Smith et al.’s idea is completely consistent with our standpoint. They stated that first, we can detect variation in epigenetic markers, second, we can choose key epigenetic biomarker(s) in regions of variance, and third, we can map these biomarker(s) to a drug response phenotype [20]. Smith et al.’s idea clearly agrees with our initial idea of a GEPh-PGx axis.

Since we found that the *TET2* gene was top, it is important to point out that it is a key player in epigenetics, hematopoiesis, and cancer biology. Its full name is Tet methylcytosine dioxygenase two, located on chromosome 4q24. TET2 is part of the TET family of enzymes, which convert 5-methylcytosine (5mC) to 5-hydroxymethylcytosine (5hmC), playing a role in DNA demethylation and epigenetic regulation. Specifically, TET2 is involved in the regulation of gene expression, stem cell differentiation, especially in hematopoiesis (formation of blood cells), immune system regulation, and epigenetic reprogramming during development. Interestingly, mutations in *TET2* are somatic (acquired) and commonly found in (1) myeloid malignancies such as myelodysplastic syndromes (MDS), acute myeloid leukemia (AML), chronic myelomonocytic leukemia (CMML); myeloproliferative neoplasms (MPNs); and (2) lymphoid cancers such as Angioimmunoblastic T-cell lymphoma (AITL) and Peripheral T-cell lymphoma (PTCL). It is also known that *TET2* mutations are among the most common in Clonal Hematopoiesis of Indeterminate Potential (CHIP), a condition where aging individuals develop hematopoietic clones without having full-blown cancer, but with an increased risk of cardiovascular disease and leukemia. Clinically, *TET2* mutations may signal different outcomes depending on the context of the disease. *TET2*-mutant cancers may respond differently to hypomethylating agents (like azacitidine or decitabine). Vitamin C (ascorbate) has been studied to enhance *TET* activity and DNA demethylation in TET2-deficient cells (preclinical). *TET2* mutations often co-occur with others (e.g., *ASXL1*, *DNMT3A*, *IDH2*), affecting disease progression and treatment [51,52].

### Limitations

The current study faces some limitations, which should be considered in similar future investigations. First of all, we used GeneCards data, which may receive updates based on novel findings in the literature. The other limitation may rely on the number of included genes, whereby future investigations would potentially generate an augmented primary gene list. Importantly, in vitro, in vivo, and clinical validations are the vital parts of testing our presented hypothesis-generating approach. More specifically, in vitro validations can be checked by expression and regulatory experiments, in vivo validations can be designed on knock down/known out of CpG-PGx SNPs in the animal of interest (mouse, rat, rabbit) and monitoring the drug’s effects on the living body; furthermore, clinical validations can be checked on individuals receiving specific drugs who performed Epigenomics or epigenetic molecular detection on the suggested CpG-PGx SNP(s). All of these validations can be widened to trans-regulation interactions of both potential CpG-PGx SNPs and possible CpG-PGx SNPs; more clearly, a forming CpG site SNP should be verified for its new positive/negative binding affinities. We believe it is plausible that CpG islands would help predict CpG-PGx SNPs in future investigations. Obviously, both clinical and real-world confirmations are highly recommended for validating our findings.

## 5. Conclusions

In conclusion, pharmacoepigenetics can provide novel insights into PGx approaches and describe complicated mechanisms involved in personalized medicine treatment options. CpG-PGx SNPs, as a conceptual framework that invites further empirical testing, can represent potential biomarkers in PGx and epigenomics, which requires more confirmation by real-world clinical findings. Based on our data, we recommend that the scientific community intensively investigate the top-scored genes reported in the current study, such as *CYP2B*, *CYP2D6*, *CYP2C19*, and *TET2*, with psychiatric and other related phenotypes. Additionally, in this study, we exposed some synonymous PGx SNPs that may be involved in CpG-PGx Disruption/Formation processes as novel clues for their impact on PGx (potential CpG-PGx SNPs). We further found other synonymous CpG-SNPs in the EMAR, confirming our primary results and, as such, highlight the uncovered roles of synonymous SNPs in regulatory mechanisms instead of functional alterations in protein structures.

## Figures and Tables

**Table 1:** Primary genes with significant PGx annotation(s) based on the PharmGKB database and their potential CpG-PGx SNP(s).

Gene	Epigenetic Process	Relevance Score	Significant PGx Annotation	CpG-PGx SNP
*CYP2B6*	Demethylation	10.40399	383	23
*CYP2C19*	Demethylation	7.372463	949	21
*CYP2D6*	Demethylation	10.74248	733	18
*COMT*	Methylation	18.34278	121	11
*CYP3A4*	Demethylation	12.93115	282	8
*FTO*	Demethylation	14.13657	11	4
*MTHFR*	Methylation	17.11379	151	3
*CYP1A2*	Demethylation	10.08261	84	2
*GRIN2B*	Methylation	22.91825	5	2
*NAT2*	Acetylation	40.88459	155	1
*TP53*	Methylation	20.33753	11	1
*MECP2*	Methylation	38.57069	3	1
*CDKN2A*	Methylation	26.32699	1	1
*DNMT3A*	Methylation	25.22683	1	1
*KAT2B*	Acetylation	25.24294	1	1
*SIN3A*	Deacetylation	5.800954	1	1
*ACSS2*	Acetylation	19.99729	2	0
*HDAC1*	Acetylation	17.49331	2	0
*MGMT*	Methylation	35.78139	1	0
*EHMT2*	Methylation	21.40185	1	0
*RASSF1*	Methylation	18.96314	1	0
*SIRT1*	Acetylation	17.80751	1	0

**Table 2:** Details of CpG-PGx SNPs found in 22 primary genes, highlighting their functions and CpG site formation/disruption.

SNP (rsID)	Gene	Function	CpG Site Situation
rs1038376	*CYP2B6*	3′UTR	Forming
rs138264188	*CYP2B6*	Missense	Disruptive
rs141666881	*CYP2B6*	Missense	Disruptive
rs142421637	*CYP2B6*	Missense	Disruptive
rs148009906	*CYP2B6*	Missense	Disruptive
rs1969136524	*CYP2B6*	Missense	Forming
rs200458614	*CYP2B6*	Missense	Disruptive
rs3181842	*CYP2B6*	3UTR	Disruptive
rs3211371	*CYP2B6*	Missense	Disruptive
rs36118214	*CYP2B6*	Intronic	Disruptive
rs373442191	*CYP2B6*	Missense	Disruptive
rs374099483	*CYP2B6*	Missense	Disruptive
rs3786547	*CYP2B6*	Intronic	Forming
rs535039125	*CYP2B6*	Missense	Disruptive
rs553968231	*CYP2B6*	Missense	Disruptive
rs58871670	*CYP2B6*	Missense	Disruptive
rs707265	*CYP2B6*	Missense	Forming
rs7246465	*CYP2B6*	3′UTR	Forming
rs750671397	*CYP2B6*	Missense	Forming
rs752695347	*CYP2B6*	Missense	Disruptive
rs764288403	*CYP2B6*	Missense	Disruptive
rs772413158	*CYP2B6*	Missense	Disruptive
rs8192709	*CYP2B6*	Missense	Disruptive
rs4244285	*CYP2C19*	Synonymous	Disruptive
rs3814637	*CYP2C19*	Intronic	Disruptive
rs183701923	*CYP2C19*	Missense	Disruptive
rs140278421	*CYP2C19*	Missense	Disruptive
rs145119820	*CYP2C19*	Missense	Disruptive
rs17878459	*CYP2C19*	Missense	Forming
rs3758581	*CYP2C19*	Missense	Forming
rs181297724	*CYP2C19*	Missense	Forming
rs118203756	*CYP2C19*	Missense	Forming
rs138142612	*CYP2C19*	Missense	Disruptive
rs72552267	*CYP2C19*	Missense	Disruptive
rs749678783	*CYP2C19*	Missense	Forming
rs764137538	*CYP2C19*	Missense	Disruptive
rs200346442	*CYP2C19*	Missense	Disruptive
rs200150287	*CYP2C19*	Missense	Disruptive
rs763625282	*CYP2C19*	Missense	Forming
rs150152656	*CYP2C19*	Missense	Disruptive
rs7902257	*CYP2C19*	Intronic	Disruptive
rs370803989	*CYP2C19*	Missense	Disruptive
rs145328984	*CYP2C19*	Missense	Disruptive
rs41291556	*CYP2C19*	Missense	Forming
rs1058172	*CYP2D6*	Missense	Disruptive
rs1065852	*CYP2D6*	Missense	Forming
rs1080985	*CYP2D6*	Missense	Forming + Disruptive
rs1080989	*CYP2D6*	Intronic	Disruptive
rs111564371	*CYP2D6*	Intronic	Forming
rs112568578	*CYP2D6*	Missense	Disruptive
rs1230912765	*CYP2D6*	Missense	Disruptive
rs138417770	*CYP2D6*	Missense	Forming + Disruptive
rs16947	*CYP2D6*	Missense	Disruptive
rs1985842	*CYP2D6*	Intronic	Forming
rs28371699	*CYP2D6*	Intronic	Disruptive
rs28371726	*CYP2D6*	Missense	Forming
rs28371738	*CYP2D6*	Intronic	Forming
rs35742686	*CYP2D6*	Frameshift	Forming
rs3892097	*CYP2D6*	Splice Acceptor	Forming
rs745746329	*CYP2D6*	Missense + Inframeshift	Disruptive
rs76187628	*CYP2D6*	Missense	Forming
rs777560972	*CYP2D6*	Missense	Disruptive
rs165599	*COMT*	3′UTR	Disruptive
rs174699	*COMT*	Intronic	Disruptive
rs2239393	*COMT*	Intronic	Forming
rs4633	*COMT*	Synonymous	Disruptive
rs4646316	*COMT*	Intronic	Disruptive
rs4680	*COMT*	Missense	Disruptive
rs5746849	*COMT*	Intronic + Enhancer	Forming
rs6269	*COMT*	3utr + CTCF	Forming
rs7287550	*COMT*	Intronic	Forming
rs740603	*COMT*	Intronic + Enhancer	Forming
rs933271	*COMT*	Intronic + Enhancer	Forming
rs1203844	*CYP3A4*	Intronic	Disruptive
rs12721627	*CYP3A4*	Missense	Forming
rs35599367	*CYP3A4*	Missense	Disruptive
rs3735451	*CYP3A4*	Intronic	Forming
rs4646437	*CYP3A4*	Missense	Disruptive
rs4986907	*CYP3A4*	Missense	Disruptive
rs4986909	*CYP3A4*	Missense	Forming
rs4986910	*CYP3A4*	Missense	Forming
rs12596638	*FTO*	Intronic	Disruptive
rs16952570	*FTO*	Intronic	Forming
rs79206939	*FTO*	Missense	Disruptive
rs9940629	*FTO*	Intronic	Forming
rs1801133	*MTHFR*	Missense	Disruptive
rs2274976	*MTHFR*	Missense	Disruptive
rs3737967	*MTHFR*	Missense	Disruptive
rs12720461	*CYP1A2*	Intronic	Disruptive
rs762551	*CYP1A2*	Intronic	Forming
rs2058878	*GRIN2B*	Intronic	Forming
rs1806201	*GRIN2B*	Synonymous	Disruptive
rs1799930	*NAT2*	Missense	Disruptive
rs1042522	*TP53*	Missense	Forming
rs1734787	*MECP2*	Intronic	Forming
rs759922342	*CDKN2A*	Missense	Forming
rs2304429	*DNMT3A*	Intronic	Disruptive
rs9829896	*KAT2B*	Intronic	Disruptive
rs7166737	*SIN3A*	Intronic	Forming

UTR means Untranslated Region.

**Table 3:** Comparison among the three best-scored genes having CpG-PGx SNPs for finding the heart of Pharmacoepigenomics.

Gene	Relevance Score	Significant PGx Annotations	Total CpG-PGx SNPs	Type of Variants	Number of CpG Site Formations	Number of CpG Site Disruptions	Indexed in PubMed
*CYP2D6*	10.74248	733	18	5	10	10	2658
*CYP2C19*	7.372463	949	21	3	7	14	2057
*CYP2B6*	10.40399	383	23	3	6	17	580

Relevance score obtained from GeneCards, PGx annotations searched in ClinPGx, and CpG sites checked in Ensembl Genome browser.

**Table 4:** Results of searching CpG site formation/disruption among remained genes based on GWAS catalog associations.

Gene	Epigenetic Process	Relevance Score	GWAS-Based Refined SNP	CpG SNP
*TET2*	Demethylation	11.1206	148	42
*JMJD1C*	Demethylation	10.36812	174	35
*HDAC9*	Deacetylation	9.43903	130	26
*KDM4B*	Demethylation	8.900465	50	22
*KDM2B*	Demethylation	8.859386	35	14
*GRIN2A*	Methylation	21.42977142	59	13
*HDAC7*	Deacetylation	7.25612	34	13
*ACAA2*	Acetylation	15.70166	52	12
*HDAC4*	Deacetylation	8.759825	34	12
*TET1*	Demethylation	12.24121	34	12
*CDH1*	Methylation	18.68703842	27	10
*KDM4C*	Demethylation	9.926583	40	10
*DNMT3B*	Methylation	25.82717514	29	9
*HDAC11*	Deacetylation	5.730898	10	8
*SLC33A1*	Acetylation	21.82475	20	7
*ACACA*	Acetylation	36.13462	17	6
*DNMT1*	Methylation	33.36149979	25	6
*KAT8*	Acetylation	15.52563	11	6
*NAGLU*	Acetylation	20.19837	6	5
*SIRT3*	Deacetylation	9.975184	23	5
*EP300*	Acetylation/Deacetylation	34.95532/8.402884	23	4
*HDAC5*	Deacetylation	7.853906	16	4
*MBD2*	Methylation	28.06098938	20	4
*PRMT5*	Methylation	19.25225449	1	1
*ACACB*	Acetylation	22.3765	12	3
*ACSS1*	Acetylation	15.36008	14	3
*AR*	Methylation	19.19925117	8	3
*HDAC2*	Acetylation/Deacetylation	14.69187/10.94646	10	3
*KDM4A*	Demethylation	11.33719	20	3
*KDM6B*	Demethylation	9.335324	11	3
*ALKBH5*	Demethylation	10.9696	10	2
*CREBBP*	Acetylation/Deacetylattion	23.61674/4.776761	7	2
*EZH2*	Methylation	17.65591049	6	2
*KDM1A*	Demethylation	15.8258	9	2
*NAT1*	Acetylation	15.67783	5	2
*RBBP7*	Deacetylation	4.671091	4	2
*TET3*	Demethylation	10.49595	19	2
*ALKBH1*	Demethylation	8.964048	4	1
*HDAC8*	Deacetylation	8.406831	3	1
*KAT2A*	Acetylation	18.43493	7	1
*KAT5*	Acetylation	21.23479	5	1
*KDM3A*	Demethylation	10.28292	2	1
*KDM6A*	Demethylation	8.683799	2	1
*MLH1*	Methylation	22.98958206	1	1
*MTA2*	Deacetylation	4.632188	3	1
*PRMT1*	Methylation	21.30334854	3	1
*SIRT6*	Deacetylation	10.88304	1	1
*TDG*	Demethylation	8.484888	1	1

Notably, the acceptable association for further checking of CpG site situations had *p* < 5 × 10^−8^) and MAF of higher than 0.05.

**Table 5:** List of possible CpG-SNPs with the highest priorities leading to disruption of a CpG site based on the remained genes of GWAS mining.

SNPS	Gene	MAF	Function	gnomAD (Aggregated) (%)
rs2984348	*HDAC8*	0.5	Enhancer	28.56
rs10849885	*KDM2B*	0.5	Synonymous/ EMAR	37.96
rs3814177	*TET1*	0.5	3′UTR	<0.01
rs86312	*NAGLU*	0.48	Missense/EMAR	46.9
rs732770	*ACACA*	0.47	Enhancer	NA
rs6087988	*DNMT3B*	0.47	TF binding/Enhancer	<0.01
rs5969751	*RBBP7*	0.47	3′UTR	NA
rs1667619	*TET3*	0.47	Synonymous/EMAR+ Enhancer	NA
rs2597512	*HDAC11*	0.45	Intronic/Enhancer	<0.01
rs112114764	*HDAC5*	0.41	Promoter	48.94
rs828867	*TET3*	0.41	3′UTR	NA
rs9929218	*CDH1*	0.4	Intronic/Enhancer	27.94
rs2107595	*HDAC9*	0.4	Enhancer/EMAR	19.8
rs2047409	*TET2*	0.39	Missense/EMAR	48.91
rs11913442	*EP300*	0.38	Promoter	55.61
rs7098181	*JMJD1C*	0.37	Intronic/EMAR	37.99
rs35291459	*HDAC4*	0.36	Synonymous	4.22
rs2301718	*TET2*	0.33	Intronic/Promoter/EMAR	23.48
rs7776786	*HDAC9*	0.32	Intronic/Enhancer	38.51
rs13389265	*HDAC4*	0.29	Intronic/Enhancer	16.54
rs2092563	*EP300*	0.25	Intronic/Enhancer	23.55
rs10426930	*KDM4B*	0.25	Intronic/EMAR/Enhancer	30.11
rs56185013	*TET2*	0.25	3′UTR	17.15
rs2288940	*DNMT1*	0.23	Intronic/CTCF/Enhancer	<0.01
rs2731338	*HDAC11*	0.22	Enhancer	72.62
rs80052686	*EZH2*	0.21	Intronic/Promoter	16.4
rs2926337	*MBD2*	0.19	Intronic/EMAR/Enhancer	25.57
rs12223627	*MTA2*	0.15	Intronic/EMAR/Enhancer	21.47
rs76714272	*ACACB*	0.14	Intronic/Enhancer	17.77
rs73015138	*DNMT1*	0.12	Intronic/Enhancer	13.31
rs11657063	*KDM6B*	0.1	EMAR	10.13
rs58644382	*HDAC4*	0.09	Enhancer	8.34
rs73107993	*HDAC7*	0.09	Intronic/CTCF	NA
rs12226402	*SIRT3*	0.09	3′UTR/EMAR/Enhancer	5.29
rs11168254	*HDAC7*	0.05	Promoter	4.37
rs41274064	*JMJD1C*	0.05	Missense/inframeshift	2.48
rs4986782	*NAT1*	0.05	Missense	1.47

MAF, EMAR, and NA refer to Minor Allele Frequency, Epigenetically Modified Accessible Region, and not available, respectively. Notably, all of the mentioned SNPs in the ClinVar database were verified to obtain any clinical relevance; however, there are just 4 SNPs in ClinVar, including rs10849885, and rs35291459, all with ACMG classification of Benign. It is noteworthy that the statistical numbers presented in this table were obtained on 19 August 2025, and they might be changed due to future updates of their sources (ClinVar, Ensembl, and gnomAD). It should be noted that the intronic variants are archived in the Supplementary Table S1, and the rationale behind this prioritization is based on the functionality of variants (structural and regulatory variants vs. intronic variants).

**Table 6:** List of possible CpG-SNPs with the maximum priorities leading to the formation of a CpG site (novel CpG site) according to the remained genes of GWAS mining.

SNPS	Gene	MAF	Func	gnomAD (Aggregated) (%)
rs1931537	*AR*	0.5	3′UTR	87.11
rs2116942	*DNMT1*	0.5	Missense	54.22
rs1935	*JMJD1C*	0.5	Missense	NA
rs7962128	*KDM2B*	0.5	3′UTR	67.31
rs7042372	*KDM4C*	0.5	Intronic/EMAR/Enhancer	36.14
rs5969750	*RBBP7*	0.5	3′UTR	NA
rs7670522	*TET2*	0.5	3′UTR	NA
rs739842	*HDAC7*	0.49	Intronic/Enhancer	69.7
rs661818	*HDAC9*	0.44	Intronic/Enhancer	NA
rs2454206	*TET2*	0.42	Missense	29.48
rs12241767	*TET1*	0.4	Missense	NA
rs609292	*HDAC5*	0.38	Intronic/Enhancer	59.02
rs7070693	*JMJD1C*	0.37	Intronic/EMAR/Enhancer	NA
rs4807687	*KDM4B*	0.37	Intronic/EMAR/Enhancer	27.83
rs9925964	*KAT8*	0.36	Splicing/Enhancer/EMAR	32.65
rs7812296	*HDAC9*	0.35	3′UTR	56.96
rs9795476	*SIRT3*	0.35	Intronic/EMAR	NA
rs34550543	*KDM4A*	0.31	Intronic/EMAR/Enhancer	NA
rs62621450	*TET2*	0.3	Missense	4.33
rs1997797	*DNMT3B*	0.29	Splicing	<0.01
rs601999	*NAGLU*	0.29	Synonymous/EMAR/Enhancer	NA
rs79491673	*MBD2*	0.27	EMAR/Enhancer	26.94
rs57917116	*KDM2B*	0.26	Intronic/EMAR/Enhancer	22.21
rs7208787	*KDM6B*	0.24	EMAR	15.87
rs7661349	*TET2*	0.24	Intronic/Promoter/EMAR	NA
rs4507125	*HDAC4*	0.22	Enhancer	NA
rs13632	*HDAC7*	0.18	3′UTR	78.95
rs591939	*NAGLU*	0.18	Synonymous/EMAR/Enhancer	17.07
rs58324296	*KDM4B*	0.16	3′UTR/EMAR	NA
rs2288937	*DNMT1*	0.12	Intronic/Enhancer	<0.01
rs71524263	*HDAC9*	0.08	Intronic/OpenChromatin/EMAR	11.44
rs41274072	*JMJD1C*	0.05	Missense	3.16
rs61031471	*KDM6B*	0.05	Missense	<0.01

MAF, EMAR, and NA refer to Minor Allele Frequency, Epigenetically Modified Accessible Region, and not available, respectively. Remarkably, we verified all of the listed SNPs in the ClinVar database to obtain any clinical relevance; however, there are just 7 SNPs in ClinVar, including rs1935, rs12241767, rs62621450, rs41274072, and rs61031471, all with ACMG classification of Benign. It should be acknowledged that the statistics presented in this table were extracted on 08/19/2025 and, as such, might be changed because of upcoming updates of their sources (ClinVar, Ensembl, and gnomAD). Notably, the intronic variants are archived in the Supplementary Table S2, and the rationale behind this ordering is because of the functionality of variants (structural and regulatory variants vs. intronic variants).

## Data Availability

Any further data will be available on a reasonable request from the corresponding author via email (alirezasharafshah@yahoo.com).

## Supplementary Materials

The following supporting information can be downloaded at: https://www.mdpi.com/article/10.3390/jpm15120579/s1, Table S1: Supplementary List of possible CpG-SNPs leading to Disruption of a CpG site based on the remained genes of GWAS mining; Table S2: Complementary list of possible CpG-SNPs with the maximum priorities leading to Formation of a CpG site (novel CpG site) according to the remained genes of GWAS mining.

## Abbreviations

The following abbreviations are used in this manuscript:

GWAS	Genome-Wide Association Studies
PGx	Pharmacogenomics
PEpGx	Pharmacoepigenomics
SNP	Single Nucleotide Polymorphism
EMAR	Epigenetically Modifiable Accessible Region
meQTLs	methylation quantitative trait loci
G-E-Ph-PGx	Genomic-Epigenomic-Phenomic-Pharmacogenomics
FDR	False Discovery Rate
MAF	Minor Allele Frequency
