# Supplementary material for: Pharmacoepigenomics in Personalized Medicine: A Hypothesis-Generating Approach to Introduce CpG-PGx SNPs as New Candidates for a Systematic Insight into Genomic-Epigenomic-Phenomic-Pharmacogenomics (G-E-Ph-PGx) Axis
Source: J Pers Med. 2025 Nov 29;15(12):579. doi: 10.3390/jpm15120579 (PMC12734362; doi:10.3390/jpm15120579)
Supplement: Supplementary file 1 [file jpm-15-00579-s001.zip › Supplementary Table S1.pdf]

Supplementary Table S1. Supplementary List of possible CpG-SNPs leading to Disruption of a CpG site based on the remained genes of GWAS mining.

SNPS	Gene	MAF	Function	gnomAD (Aggregated) (%)
**rs13245206**	*HDAC9*	0.5	Intronic	45.17
**rs10237149**	*HDAC9*	0.5	Intronic	42.58
**rs6951745**	*HDAC9*	0.5	Intronic	44
**rs12001316**	*KDM4C*	0.5	Intronic	NA
**rs9884984**	*TET2*	0.5	Intronic	NA
**rs6533183**	*TET2*	0.5	Intronic	60.27
**rs742630**	*DNMT3B*	0.49	Intronic	55.91
**rs1569686**	*DNMT3B*	0.49	Intronic	NA
**rs13242758**	*HDAC9*	0.49	Intronic	42.85
**rs10995489**	*JMJD1C*	0.49	Intronic	58.42
**rs2610528**	*MBD2*	0.49	Intronic	<0.01
**rs10010325**	*TET2*	0.49	Intronic	49.75
**rs2903385**	*TET2*	0.49	Intronic	49.53
**rs11097882**	*TET2*	0.49	Intronic	47.38
**rs6083730**	*ACSS1*	0.48	Intronic	<0.01
**rs756854**	*HDAC9*	0.48	Intronic	46.9
**rs4982708**	*PRMT5*	0.48	Intronic	45.95
**rs6441017**	*SLC33A1*	0.48	Intronic	45.95
**rs2647243**	*TET2*	0.48	Intronic	91.43
**rs4903626**	*ALKBH1*	0.47	Intronic	56.58
**rs1978485**	*KAT8*	0.47	Intronic	57.66
**rs17693103**	*NAT1*	0.47	Intronic	0.19
**rs10237280**	*HDAC9*	0.46	Intronic	42.13
**rs1057199**	*KDM4C*	0.46	Intronic	53.51
**rs12700003**	*HDAC9*	0.45	Intronic	49.31
**rs2613765**	*KDM4B*	0.45	Intronic	42.44
**rs5918763**	*AR*	0.45	Intronic	86.7
**rs910527**	*ACSS1*	0.44	Intronic	58.42
**rs59735493**	*KAT8*	0.43	Intronic	36.08
**rs9789310**	*KDM4B*	0.43	Intronic	36.9
**rs7663401**	*TET2*	0.43	Intronic	52.76
**rs717388**	*HDAC2*	0.42	Intronic	NA
**rs6479901**	*JMJD1C*	0.42	Intronic	67.44
**rs28725459**	*KAT8*	0.42	Intronic	34.9
**rs2664419**	*TET1*	0.42	Intronic	58.95
**rs62332762**	*TET2*	0.42	Intronic	29.28
**rs10822145**	*JMJD1C*	0.41	Intronic	44.65
**rs2613786**	*KDM4B*	0.41	Intronic	53.33

**rs2680392**	*ACACA*	0.4	Intonic	66.29
**rs60920123**	*GRIN2A*	0.4	Intronic	39.82
**rs12983032**	*KDM4B*	0.4	Intronic	NA
**rs11721948**	*TET2*	0.4	Intronic	25.64
**rs7895472**	*JMJD1C*	0.39	Intronic	43.65
**rs10822168**	*JMJD1C*	0.39	Intronic	43.52
**rs10995527**	*JMJD1C*	0.39	Intronic	<0.01
**rs10761723**	*JMJD1C*	0.39	Intronic	NA
**rs10822161**	*JMJD1C*	0.39	Intronic	43.36
**rs2007403**	*TET2*	0.39	Intronic	49.27
**rs7215365**	*ACACA*	0.38	Intonic	NA
**rs7077580**	*JMJD1C*	0.38	Intronic	42.6
**rs7084707**	*JMJD1C*	0.37	Intronic	NA
**rs61320757**	*KAT8*	0.37	Intronic	30.16
**rs7902343**	*JMJD1C*	0.36	Intronic	40.71
**rs2047408**	*TET2*	0.35	Intronic	NA
**rs12450937**	*ACACA*	0.34	Intonic	49.48
**rs1040553**	*DNMT3B*	0.34	Intronic	50.13
**rs993419**	*DNMT3B*	0.34	Intronic	50.18
**rs9922678**	*GRIN2A*	0.34	Intronic	37.55
**rs17139675**	*HDAC9*	0.34	Intronic	23.73
**rs11065589**	*KDM2B*	0.34	Intronic	60.46
**rs4780790**	*GRIN2A*	0.33	Intronic	67.43
**rs1543158**	*NAGLU*	0.33	Intronic	31.23
**rs9994426**	*TET2*	0.31	Intronic	67.41
**rs28758996**	*KDM2B*	0.3	Intronic	37.13
**rs12578785**	*KDM2B*	0.3	Intronic	33.63
**rs28663167**	*KDM2B*	0.3	Intronic	NA
**rs10998287**	*TET1*	0.3	Intronic	32.91
**rs56389811**	*HDAC7*	0.29	Intronic	0.02
**rs8055912**	*CDH1*	0.28	Intronic	NA
**rs8056338**	*CDH1*	0.28	Intronic	<0.01
**rs34574947**	*HDAC9*	0.28	Intronic	32.2
**rs197152**	*KDM4B*	0.28	Intronic	NA
**rs368328**	*HDAC5*	0.27	Intronic	68.85
**rs10998288**	*TET1*	0.27	Intronic	28.48
**rs12449696**	*ACACA*	0.26	Intonic	21.13
**rs6511611**	*DNMT1*	0.26	Intronic	NA
**rs7250055**	*KDM4B*	0.25	Intronic	24.68
**rs12774282**	*TET1*	0.25	Intronic	8.74
**rs2647249**	*TET2*	0.25	Intronic	81.36
**rs6507940**	*ACAA2*	0.24	Intronic	29.01
**rs11869205**	*ALKBH5*	0.24	Intronic	NA

**rs7699743**	*TET2*	0.24	Intronic	69.55
**rs130017**	*CREBBP*	0.23	Intronic	17.75
**rs1178102**	*HDAC9*	0.23	Intronic	19.74
**rs684214**	*NAGLU*	0.23	Intronic	19.95
**rs1178331**	*HDAC9*	0.22	Intronic	NA
**rs6969316**	*HDAC9*	0.22	Intronic	23.65
**rs2647261**	*TET2*	0.22	Intronic	26.11
**rs9304383**	*ACAA2*	0.21	Intronic	19.42
**rs10415880**	*PRMT1*	0.21	Intronic	29.05
**rs390677**	*SLC33A1*	0.21	Intronic	20.67
**rs382534**	*SLC33A1*	0.21	Intronic	20.54
**rs112013645**	*SLC33A1*	0.21	Intronic	20.89
**rs59103188**	*ACAA2*	0.2	Intronic	10.89
**rs5758223**	*EP300*	0.2	Intronic	71.5
**rs11153476**	*HDAC2*	0.2	Intronic	NA
**rs13247375**	*HDAC9*	0.2	Intronic	18.88
**rs7896910**	*JMJD1C*	0.2	Intronic	23.36
**rs72837033**	*JMJD1C*	0.2	Intronic	NA
**rs4135054**	*TDG*	0.2	Intronic	17.29
**rs6533185**	*TET2*	0.2	Intronic	12.85
**rs12600694**	*ALKBH5*	0.19	Intronic	20.61
**rs59022814**	*HDAC4*	0.17	Intronic	7.08
**rs6706275**	*HDAC4*	0.15	Intronic	24.38
**rs34386000**	*TET1*	0.15	Intronic	14.93
**rs66487118**	*DNMT1*	0.14	Intronic	22.11
**rs33938520**	*KDM4B*	0.14	Intronic	21.19
**rs34009962**	*KDM4B*	0.14	Intronic	21.41
**rs75792932**	*KDM4B*	0.13	Intronic	19.56
**rs10420726**	*KDM4B*	0.13	Intronic	19.67
**rs12965923**	*ACAA2*	0.12	Intronic	NA
**rs28550823**	*GRIN2A*	0.12	Intronic	18.43
**rs17140133**	*HDAC9*	0.12	Intronic	9.08
**rs60186830**	*KDM2B*	0.12	Intronic	9.29
**rs8110642**	*KDM4B*	0.12	Intronic	92.21
**rs35098345**	*TET2*	0.12	Intronic	12.31
**rs12926704**	*GRIN2A*	0.11	Intronic	11.11
**rs6900394**	*HDAC2*	0.11	Intronic	<0.01
**rs75888251**	*JMJD1C*	0.11	Intronic	4.03
**rs116438504**	*JMJD1C*	0.11	Intronic	13.33
**rs76698003**	*KDM2B*	0.11	Intronic	5.5
**rs183716438**	*ACAA2*	0.1	Intronic	8.22
**rs11984041**	*HDAC9*	0.1	Intronic	12.11
**rs6958814**	*HDAC9*	0.1	Intronic	8.54

**rs35570603**	*TET1*	0.1	Intronic	8.57
**rs11168251**	*HDAC7*	0.09	Intronic	16.21
**rs113161209**	*EZH2*	0.08	Intronic	6.48
**rs7959510**	*HDAC7*	0.08	Intronic	84.51
**rs73107980**	*HDAC7*	0.08	Intronic	16.02
**rs2675228**	*HDAC11*	0.08	Intronic	89.77
**rs2675231**	*HDAC11*	0.08	Intronic	89.72
**rs17035323**	*TET2*	0.08	Intronic	13.13
**rs59050225**	*JMJD1C*	0.07	Intronic	3.59
**rs79109558**	*KDM4C*	0.06	Intronic	2.9
**rs111690247**	*KDM2B*	0.05	Intronic	6.02

MAF and NA refer to Minor Allele Frequency and not available, respectively. Notably, all of the mentioned SNPs in ClinVar database were verified to obtain any clinical relevance; however, there are just 4 SNPs in ClinVar including rs2647243, and rs382534 all with ACMG classification of Benign. It is noteworthy that the statistical numbers presented in this table obtained on 08/19/2025 and they might be changed due to future updates of their sources (ClinVar, Ensembl, and gnomAD).
