# Supplementary material for: Pharmacoepigenomics in Personalized Medicine: A Hypothesis-Generating Approach to Introduce CpG-PGx SNPs as New Candidates for a Systematic Insight into Genomic-Epigenomic-Phenomic-Pharmacogenomics (G-E-Ph-PGx) Axis
Source: J Pers Med. 2025 Nov 29;15(12):579. doi: 10.3390/jpm15120579 (PMC12734362; doi:10.3390/jpm15120579)
Supplement: Supplementary file 1 [file jpm-15-00579-s001.zip › Supplementary Table S2.pdf]

Supplementary Table S2. Complementary list of possible CpG-SNPs with the maximum priorities leading to Formation of a CpG site (novel CpG site) according to the remained genes of GWAS mining.

**SNPS**	**Gene**	**MAF**	**Func**	**gnomAD (Aggre-gated) (%)**
rs6489811	*KDM2B*	0.5	Intronic	56.58
rs2613766	*KDM4B*	0.5	Intronic	NA
rs960658	*KDM4C*	0.5	Intronic	57.1
rs7037266	*KDM4C*	0.5	Intronic	NA
rs9884296	*TET2*	0.5	Intronic	65.38
rs5952279	*KDM6A*	0.5	Intronic	NA
rs4827402	*AR*	0.49	Intronic	NA
rs10995505	*JMJD1C*	0.49	Intronic	NA
rs62647699	*KDM4B*	0.49	Intronic	NA
rs9876116	*MLH1*	0.49	Intronic	0
rs10193548	*HDAC4*	0.48	Intronic	NA
rs6658300	*KDM4A*	0.48	Intronic	NA
rs8089411	*MBD2*	0.48	Intronic	41.62
rs7616853	*SLC33A1*	0.48	Intronic	41.1
rs9949052	*ACAA2*	0.47	Intronic	NA
rs9676981	*KDM4B*	0.47	Intronic	40.84
rs6794232	*SLC33A1*	0.47	Intronic	39.79
rs10237366	*HDAC9*	0.46	Intronic	<0.01
rs17429745	*TET2*	0.46	Intronic	NA
rs62331124	*TET2*	0.46	Intronic	NA
rs10998356	*TET1*	0.45	Intronic	48.76
rs1097784	*GRIN2A*	0.44	Intronic	45.51
rs4852018	*HDAC4*	0.44	Intronic	NA
rs9646283	*CDH1*	0.43	Intronic	28.52
rs9415676	*JMJD1C*	0.43	Intronic	43.89
rs11865499	*KAT8*	0.43	Intronic	35.99
rs4911257	*DNMT3B*	0.42	Intronic	NA
rs2647239	*TET2*	0.42	Intronic	NA
rs2466920	*TET2*	0.42	Intronic	NA
rs12150830	*ACAA2*	0.41	Intronic	NA
rs10761765	*JMJD1C*	0.41	Intronic	NA
rs1868289	*GRIN2A*	0.4	Intronic	NA
rs10822163	*JMJD1C*	0.39	Intronic	<0.01
rs7923609	*JMJD1C*	0.39	Intronic	43.42
rs10822160	*JMJD1C*	0.39	Intronic	0.02

rs7095571	*JMJD1C*	0.39	Intronic	42.96
rs10761771	*JMJD1C*	0.39	Intronic	42.98
rs4405189	*JMJD1C*	0.39	Intronic	43.03
rs10444491	*KDM2B*	0.39	Intronic	38.35
rs7031625	*KDM4C*	0.38	Intronic	NA
rs7683416	*TET2*	0.38	Intronic	45.4
rs2285657	*KAT2A*	0.37	Intronic	35.48
rs35158985	*CDH1*	0.36	Intronic	36.32
rs34770920	*ACAA2*	0.36	Intronic	63.76
rs8093891	*ACAA2*	0.35	Intronic	NA
rs1900101	*ACACA*	0.35	Intonic	28.99
rs7201930	*GRIN2A*	0.35	Intronic	39.19
rs10761737	*JMJD1C*	0.35	Intronic	35.67
rs9414802	*JMJD1C*	0.35	Intronic	27.48
rs710956	*KDM4B*	0.34	Intronic	NA
rs2647234	*TET2*	0.34	Intronic	32.54
rs9964304	*ACAA2*	0.33	Intronic	31.88
rs7190785	*GRIN2A*	0.33	Intronic	64.22
rs169080	*KDM4B*	0.33	Intronic	66.24
rs7191183	*GRIN2A*	0.32	Intronic	35.13
rs8088929	*ACAA2*	0.31	Intronic	67.22
rs7307046	*HDAC7*	0.31	Intronic	61.67
rs7191999	*GRIN2A*	0.3	Intronic	NA
rs3791452	*HDAC4*	0.3	Intronic	68.91
rs4758633	*SIRT3*	0.3	Intronic	NA
rs10902106	*SIRT3*	0.3	Intronic	63.43
rs28608872	*CDH1*	0.28	Intronic	29
rs7972177	*HDAC7*	0.28	Intronic	28.36
rs10975974	*KDM4C*	0.28	Intronic	9.2
rs10022109	*TET2*	0.28	Intronic	21.35
rs10744776	*ACACB*	0.27	Intronic	78.32
rs9646284	*CDH1*	0.27	Intronic	NA
rs2424905	*DNMT3B*	0.27	Intronic	58.72
rs137993948	*KDM1A*	0.27	Intronic	14.19
rs1023430	*SIRT3*	0.27	Intronic	19.44
rs350844	*SIRT6*	0.27	Intronic	85.92
rs904274	*TET2*	0.27	Intronic	<0.01
rs2072945	*KDM1A*	0.26	Intronic	NA
rs13103161	*TET2*	0.26	Intronic	29.01
rs2011779	*CDH1*	0.25	Intronic	31.19
rs4420522	*CDH1*	0.24	Intronic	26.66
rs2526639	*HDAC9*	0.24	Intronic	NA
rs28540102	*KDM4B*	0.24	Intronic	NA

rs1654885	*ACACB*	0.23	Intronic	83.17
rs56137247	*HDAC4*	0.23	Intronic	NA
rs11726786	*TET2*	0.23	Intronic	25.43
rs2133084	*TET2*	0.23	Intronic	84.85
rs6533181	*TET2*	0.23	Intronic	73.96
rs6087992	*DNMT3B*	0.22	Intronic	63.33
rs3791478	*HDAC4*	0.22	Intronic	20.17
rs2030057	*TET1*	0.22	Intronic	NA
rs11168236	*HDAC7*	0.21	Intronic	15.55
rs75601653	*KAT5*	0.21	Intronic	NA
rs407258	*SLC33A1*	0.21	Intronic	20.64
rs1977825	*TET1*	0.21	Intronic	21.64
rs28628339	*CDH1*	0.2	Intronic	<0.01
rs7510675	*EP300*	0.2	Intronic	71.58
rs4760624	*HDAC7*	0.2	Intronic	NA
rs302177	*HDAC9*	0.2	Intronic	NA
rs2393967	*JMJD1C*	0.2	Intronic	23.41
rs4832290	*KDM3A*	0.2	Intronic	<0.01
rs2523162	*HDAC5*	0.19	Intronic	75.33
rs13243921	*HDAC9*	0.19	Intronic	NA
rs2620832	*KDM4B*	0.19	Intronic	NA
rs6818511	*TET2*	0.19	Intronic	75.8
rs3791033	*KDM4A*	0.18	Intronic	25.6
rs7499643	*CREBBP*	0.17	Intronic	NA
rs78628688	*KDM2B*	0.17	Intronic	8.64
rs10010512	*TET2*	0.17	Intronic	18.86
rs7896294	*JMJD1C*	0.16	Intronic	<0.01
rs12352785	*KDM4C*	0.16	Intronic	74.86
rs61393039	*HDAC9*	0.15	Intronic	NA
rs10975917	*KDM4C*	0.13	Intronic	NA
rs11662691	*ACAA2*	0.12	Intronic	13.39
rs34149349	*HDAC7*	0.12	Intronic	<0.01
rs2894069	*TET1*	0.12	Intronic	29.03
rs7206296	*GRIN2A*	0.09	Intronic	<0.01
rs13337187	*GRIN2A*	0.09	Intronic	14.02
rs12250472	*JMJD1C*	0.09	Intronic	8.29
rs75321784	*TET2*	0.09	Intronic	11.38
rs17430251	*TET2*	0.09	Intronic	11.7
rs1549349	*KDM2B*	0.08	Intronic	NA
rs138578374	*HDAC4*	0.07	Intronic	0.08
rs2630452	*HDAC11*	0.07	Intronic	90.26
rs2655232	*HDAC11*	0.07	Intronic	NA
rs62115563	*KDM4B*	0.07	Intronic	11.35

rs2675229	*HDAC11*	0.06	Intronic	90.93
rs77074018	*HDAC11*	0.05	Intronic	3.84

MAF and NA refer to Minor Allele Frequency, Epigenetically Modified Accessible Region, and not available, respectively. Remarkably, we verified all of the listed SNPs in ClinVar database to obtain any clinical relevance; however, there are just 7 SNPs in ClinVar including rs2072945, and rs4832290 all with ACMG classification of Benign. It should be acknowledged that the statistics presented in this table was extracted on 08/19/2025 and as such might be changed because of upcoming updates of their sources (ClinVar, Ensembl, and gnomAD).
